# Secondarily estimated cure fraction and five-year recurrence-free conditional survival probabilities among patients undergoing surgical resection for hepatocellular carcinoma presenting with minor gross vascular invasion

**DOI:** 10.1186/s12957-021-02331-1

**Published:** 2021-07-26

**Authors:** Byungje Bae, Sung Kyu Song, Eunyoung Choi, Chul-Woon Chung, Yongkeun Park

**Affiliations:** 1 Department of Surgery, Catholic Kwandong University International St. Mary’s Hospital, Catholic Kwandong University College of Medicine, 25 Simgokro 100gil, Seo-gu, 22711 Incheon, Korea; 2 Catholic Kwandong University College of Medicine, Incheon, Korea

**Keywords:** Cure model, Hepatectomy, Portal vein tumor thrombus, Survival analysis

## Abstract

**Background:**

Surgical resection (SR) has been selectively applied in hepatocellular carcinoma (HCC) presenting with minor gross vascular invasion (mGVI) which is defined when tumor invasion is confined to second-order portal branches or segmental branches of hepatic vein. However, little data of long-term outcomes are available for supporting the role of SR as a potentially curable therapeutic option for HCC presenting with mGVI. This study is aimed to estimate a statistical cure fraction and the improvement of recurrence-free conditional survival (RFCS) over time among patients undergoing SR for HCC presenting with mGVI.

**Methods:**

The literature search was conducted focusing on previous studies that investigated the long-term survival rates of patients after SR for HCC presenting with mGVI. The reference cohort was extracted from a study including patients undergoing SR for HCC without vascular invasion. A non-mixture cure model was adopted to estimate the statistical cure fraction. The 5-year RFCS probabilities were also calculated.

**Results:**

Three retrospective studies were secondarily analyzed. The probability of being statistically cured after SR for HCC presenting with mGVI was 7.3% (95% confidence interval, 4.4%–11.2%) in the mGVI group, lower than that of the reference cohort (hazard ratio, 1.81; 95% confidence interval, 1.59–2.05). The estimated 5-year RFCS probabilities improved with each additional year of survival. Moreover, 1 year after SR, the 5-year RFCS probabilities of patients with HCC presenting with mGVI was essentially the same as that of the reference cohort.

**Conclusions:**

This study shows that a cure can be expected in around seven percent of patients undergoing SR for HCC presenting with mGVI. Furthermore, recurrence-free survival expectancy improves dramatically over time among those patients who do not have recurrence. Overall, these findings suggest that SR should be considered as a potentially curable treatment for patients with HCC presenting with mGVI.

## Background

Hepatocellular carcinoma (HCC) has been receiving a growing attention and is a commonly diagnosed cancer throughout the world [1]. Epidemiological studies show that both the incidence rate and related mortality of HCC increased during the last decade [2]. Recently, many studies have been performed to elucidate molecular mechanisms of HCC, and to identify an effective genetic biomarkers for individualized treatment [3–6]. Curative treatment options, such as surgical resection (SR), liver transplantation (LT), and locoregional therapies, provide relatively favorable outcomes for patients fulfilling their own selective criteria [7–9]. Among the known treatment options, SR remains the gold standard for early-stage HCC, especially for the treatment of solitary tumor with the preservation of good liver function, whereas LT is reserved for early-stage HCC patients with a history of liver cirrhosis. However, it is still difficult to treat patients with HCC considering that many patients are diagnosed at the intermediate or advanced stage and that they are usually recommended to undergo palliative therapies [7].

The presence of gross vascular invasion (GVI) (mostly portal vein tumor involvement) at initial presentation reflects advanced tumor stage and predicts a poor prognosis in patients with HCC. The prevalence of GVI which can be identified during radiological imaging has been reported to be 35% in newly detected HCC [10]. In spite of this, the optimal treatment strategy for this distinct stage remains inconclusive. The current Barcelona Clinic for Liver Cancer staging system recommends a systemic therapy with sorafenib at this stage, but the outcome has not been promising for patients with HCC presenting with GVI [11]. Recent studies by experienced surgeons asserted that SR provided superior treatment outcomes compared to other treatment modalities, such as transarteiral chemoembolization or sorafenib, for patients with HCC presenting with GVI [12, 13]. Systemic reviews and meta-analyses supporting the outcomes of these studies were also found [14]. Furthermore, it would not be appropriate to determine therapeutic modalities under the supposition that all HCC presenting with GVI are of the same status or stage. The survival outcomes after SR for HCC with GVI were different depending on the extent of GVI [12]. Minor gross vascular invasion (mGVI) confined to second-order branches is usually associated with better survival than GVI involving the first-order branches or the main portal trunk [15]. Therefore, SR could be more as a therapeutic option in patients with HCC presenting with mGVI.

However, physicians are still hesitant to apply a surgical approach to HCC presenting with mGVI, even in patients with a well preserved liver function. This may be because the prognosis following SR for HCC presenting with mGVI has been reported worse compared with that for HCC without GVI. So, supporting evidence should be given for holding the opinion that SR is a valuable treatment modality for HCC presenting with mGVI. At this point, the following questions can be raised: Is HCC presenting with mGVI curable by SR? And, is mGVI still a deteriorating factor for the future survival even in long-term recurrence free survivors following SR? If a portion of patients can be cured, or if the prognosis becomes similar between patients with HCC presenting with mGVI and those without GVI after long-term periods of recurrence free survival, the value of SR for HCC with mGVI could increase. Unfortunately, conventional methods of survival estimates are not suitable to show evidence regarding these issues, which inevitably necessitates the use of new statistical methods to provide insights into curability and the changes in prognosis over time.

The concepts of the cure model and conditional survival (CS) probability are suitable alternatives of addressing these issues. A cure model has been used to estimate the cure fraction, defined as the proportion of statistically cured patients. A statistical cure is said to occur when the survival curves reach a plateau at the end [16]. Not only cure fractions, but also ‘time to cure’ can be obtained from the cure model [17]. CS probability has been also used to describe the dynamic possibility of survival, taking account of the changes of death risk with time lapsing. It is defined as the probability of surviving an additional period conditional on being alive at a defined time point, and can be easily implemented in clinical research [18]. The aims of this study are (1) to estimate what proportion of patients can be cured after SR for HCC presenting with mGVI, and (2) to determine the changes of recurrence-free survival expectancy of those patients with time lapsing. The findings were compared to corresponding results obtained in patients with HCC without GVI. Cure fractions, time to cure, and CS probabilities were secondarily analyzed and estimated from the survival data of the originally published studies.

## Methods

### Literature search

Further research was performed in electronic databases (MEDLINE, Cochrane library, KMBase, and KoreaMed) from their date of inception to December 2020. To obtain the maximum sensitivity of the search strategy and effectively identify all studies, we paired the term “hepatocellular carcinoma” with the following Medical Subject Headings terms or keywords: “macrovascular invasion,” “gross vascular invasion,” “portal vein invasion,” “portal vein tumor thrombus,” “hepatic vein invasion,” “hepatic vein tumor thrombus,” “partial hepatectomy,” “surgery,” “surgical procedure,” or “surgical resection.” Titles and abstracts of retrieved articles were also examined to exclude irrelevant reports. Review articles were also reviewed in order to find potentially relevant studies. Moreover, all publications were limited in the English language. For further screening, all selected articles were systematically assessed using the inclusion and exclusion criteria by two independent investigators (BB and SKS).

### Eligibility criteria

Eligible studies in which patient cohorts underwent open or laparoscopic primary surgery for HCC accompanied by GVI were included; these studies also analyzed the potential association between the prognosis of HCC patients and GVI. The recurrence-free survival was the outcome measure between patients with or without GVI and the extent of GVI. The extent of GVI was re-classified as Vp0 ~ Vp4 or Vv0 ~ Vv3, respectively, according to the General Rules for the Study of Primary Liver Cancer by the Korean Liver Cancer Study Group [19]. In this study, Vp1 ~ 2 and Vv1 were defined as mGVI. Therefore, cases in which the extent of GVI involved the major portal or hepatic vein branches (Vp3 ~ 4 or Vv2 ~ 3) were excluded. Eligibility required reports of recurrence-free survival probabilities more than 5 years following primary SR, which included figures of Kaplan–Meier (KM) survival curves. Studies showing the survival outcomes equal or less than 5 years postoperatively were excluded. In order to compare the difference in the cure fractions according to the presence or absence of mGVI, a recent published article reporting the long-term recurrence-free survival and their cure fraction of patients with HCC without GVI was included as a reference cohort.

### Survival data extraction and reconstruction of Kaplan–Meier data

We reconstructed the KM survival data from the published survival curves. The time and survival probability coordinates were extracted from the figures of survival curves using the DigitizeIt software (www.digitizeit.de). We extracted the numbers of patients at risk and the total numbers of events from the text, when available. Pseudo-individual patient data (IPD) were generated using a unique algorithm introduced by Guyot and colleagues that was adopted to inversely solve the KM equations [20].

### Non-mixture cure model in analyzing long-term survivors

Cure models have been used with the basic premise that a certain portion of patients will never face the event of interest, such as disease-specific mortality. It may be particularly appealing to oncologists who believe that a substantial fraction of cancer patients will survive without relapse. This concept can be defined as the cure fraction. What should be noted here is that the estimation of cure is performed at a population level. Cure models interpret cure as occurring when the survival time tends to infinite and herein time to cure was assessed. So, time to cure is defined as the minimum time a patient must survive before being assessed for the possible presence of a cure. In this study, we applied the non-mixture cure model to identify the proportion of patients who can be considered as being cured. The non-mixture cure model is a parametric cure model that estimates an asymptote for the survival function at the cure proportion, chosen for its applicability in tumor recurrence modeling [17].

### Calculation of the conditional survival (CS) probabilities

The conditional probability of A given B is the probability of event A, updated on the basis of the knowledge that the event B occurred, which is denoted by P(A|B) = P(A ∩ B)P(B). With the same concept, the CS probability is defined as the probability of patients to survive for an additional period, considering that those patients have already survived for a defined period of time [21]. The CS probabilities in this study have been estimated based on the Kaplan–Meier cumulative survival data. S(t) denotes the survival probability at a specific time “t” in the actuarial life table, whereas CS probability is calculated as CS (y|x) = S(x + y)/S(x). For example, the CS probability of a patient (who has already survived 3 years) of surviving an additional 5 year, S(5/3), is adjusted by dividing the 5-year actuarial life table survival estimate, S(8), by the 3-year survival estimate, S(3); thus, S(5/3) = S(3 + 5)/S(3) = S(8)/S(3).

### Statistical analysis

All statistical analyses were performed using R (version 3.6.3; The R Foundation for Statistical Computing, Vienna, Austria). For survival analysis, HR calculation, and plotting of survival graph, “ggkm” and “survival” package in R was used. We also used another package “flexsurvcure” in R for the non-mixture cure model analysis [22]. We gathered the CS probabilities at each time period. The CS differences observed between subgroups were compared with the calculation of standardized differences. In the case of two proportions, P1 and P2, the standardized difference is calculated as (P2-P1) divided by square root of aP(1-aP), where aP is the mean of P1 and P2: d values lower than |0.1| indicate very small differences between proportions; d values between |0.1| and |0.3| indicate small differences, d values between |0.3| and |0.5| indicate moderate differences, and d values greater than |0.5| indicate considerable differences [23].

## Results

### Study selection and characteristics

**Fig. 1:**
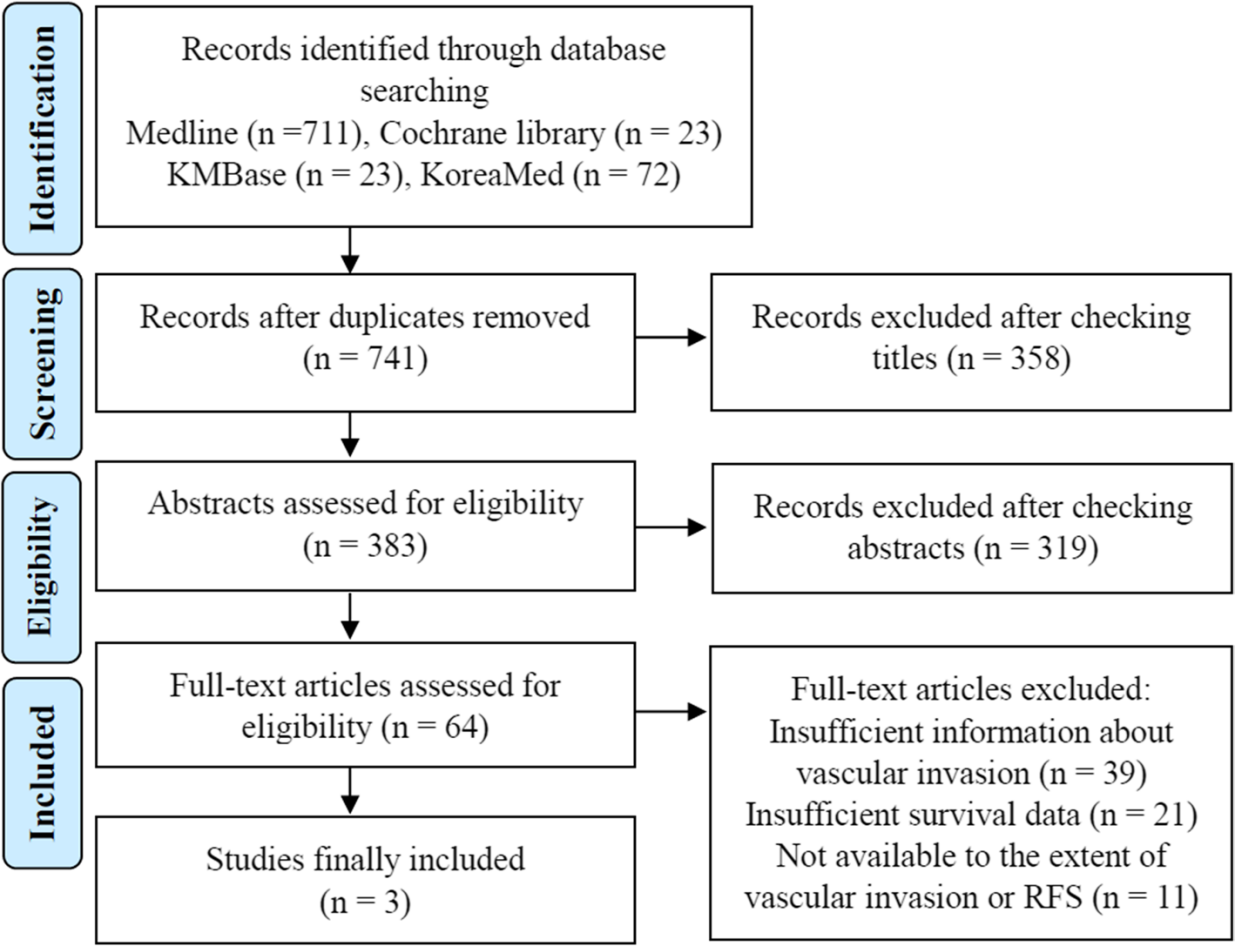
Flow chart for study selection. RFS, recurrence-free survival

Initially, we assembled a total of 741 articles from the electronic databases, excluding the duplicates. 677 articles were excluded after screening the titles and abstracts. Then, we reviewed the full texts of the remaining 64 articles, of which 39 studies were excluded because they did not focus on GVI but other pathologic or molecular biomarkers. 21 studies were excluded for lacking sufficient survival data to estimate cure rates or CS probabilities. 8 studies were excluded because GVI was not divided according to its extent, and 3 studies were excluded for only having overall survival data of patients (no long-term recurrence free survival data of patients). Finally, three studies met the selection criteria and were included in our analysis [15, 24, 25]. Fig. 1 shows a flow diagram summarizing the study selection process. The detailed characteristics and baseline demographics of the patients in the selected studies are presented in Table 1. The publication years of the studies were from 2010 to 2017, originating from China, Japan, and South Korea, respectively. The survival data of all three study was based on each single center registry. A total of 333 patients were enrolled in this data analysis. To compare the difference in the cure fractions according to the presence or absence of mGVI, a recent published study was identified as a reference cohort. It was a multicenter based retrospective cohort study that reported the long-term recurrence-free survival and their cure fraction of patients undergoing SR for HCC without GVI [26].

**Table 1:** Characteristics and baseline demographics of the patients in each included study

Author (published year)	Study design (country)	Study period	Median follow-up period (months)	No. of pts	Gender (M/F)	Ages (years)	Etiology (HBV/others)	AFP (ng/ml)	Resection type (minor/major)	Tumor (S/M)	Tumor size (cm)	CP (A/B)	Cirrhosis (N/Y)
mGVI cohorts													
Shi J, et al. (2010) [15]	RC (China)	2001–2003	6.4 (3–72)	139	123/16	48.3 ± 9.7	131/8	635.2 ± 436.3	38/91	14/10	16/123 (< 5/ ≥ 5)	134/5	28/111
Yamamoto Y, et al. (2015) [24]	RC (Japan)	1980–2009	50.3	73	67/6	60.1 ± 9.7	NA	9006 ± 34,819	NA	41/32	5.47 ± 3.38	NA	NA
Park YK, et al. (2017) [25]	RC (Korea)	1994–2012	40 (1–204)	103	85/18	51.4 ± 10.5	83/20	7336.6 ± 16,371.8	46/57	76/27	7.2 ± 3.7	94/8	47/46
Reference cohort													
Cucchetti A, et al. (2020) [26]	RC (6 centers)	1990–2015	NA	2,523	2,063/460	median (IQR), 60 (50–68)	1,394/1,129	median (IQR), 21.5 (5.6–300)	NA	1,948/575	median (IQR), 4.0 (2.5–6.2)	2,414/109	NA

*CP* Child Pugh classification, *IQR* Interquartile range, *M/F* Male/female, *mGVI* Minor gross vascular invasion, *NA* Not available, *Pts*. Patients, *RC* Retrospective cohort, *S/M* Solitary/multiple

### Cure fractions according to the presence or absence of mGVI

**Fig. 2:**
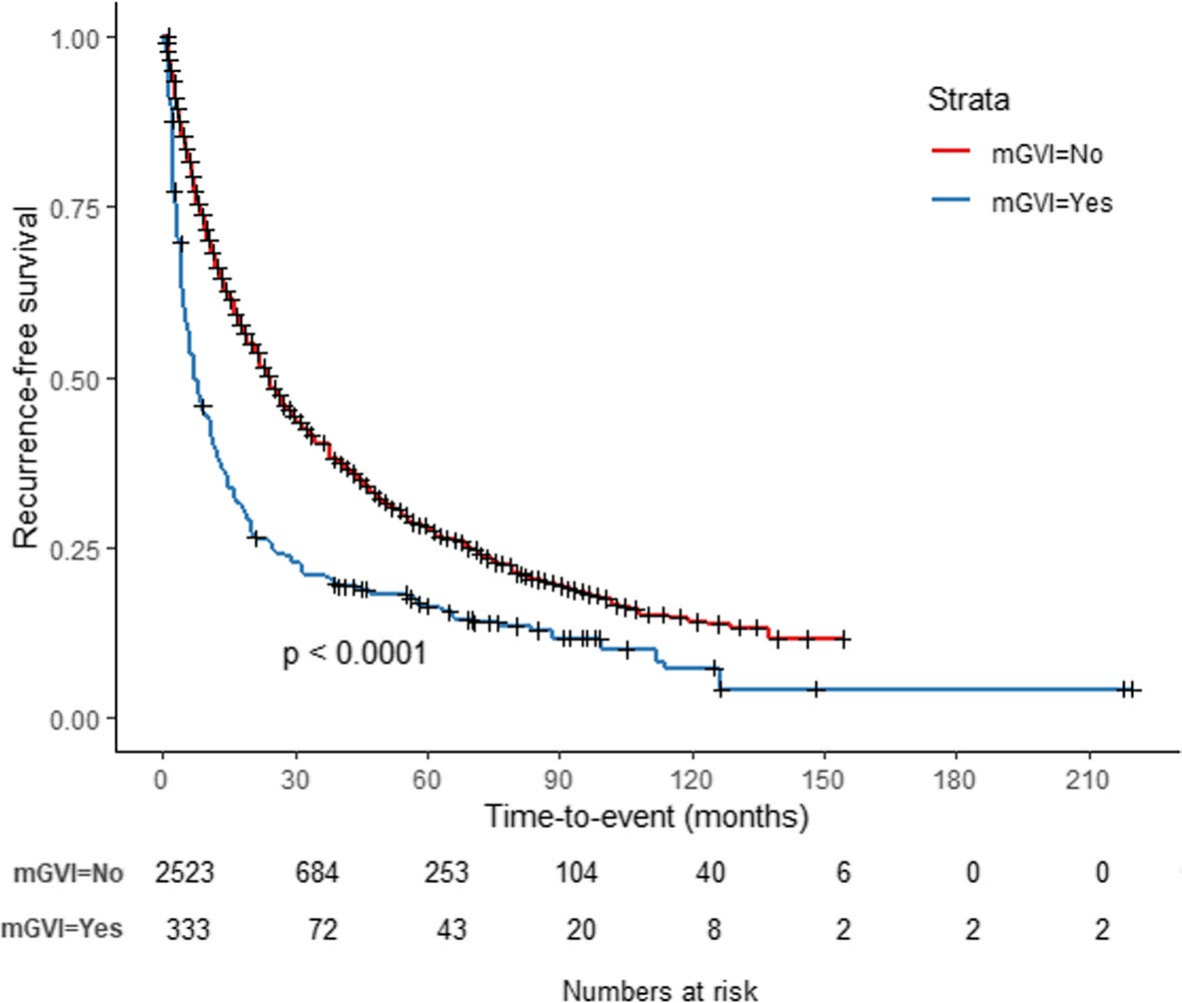
Reconstructed Kaplan–Meier (KM) survival curves among two groups according to minor gross vascular invasion (mGVI) secondarily from original KM data using the algorithm of Guyot P et al. [13]

**Fig. 3:**
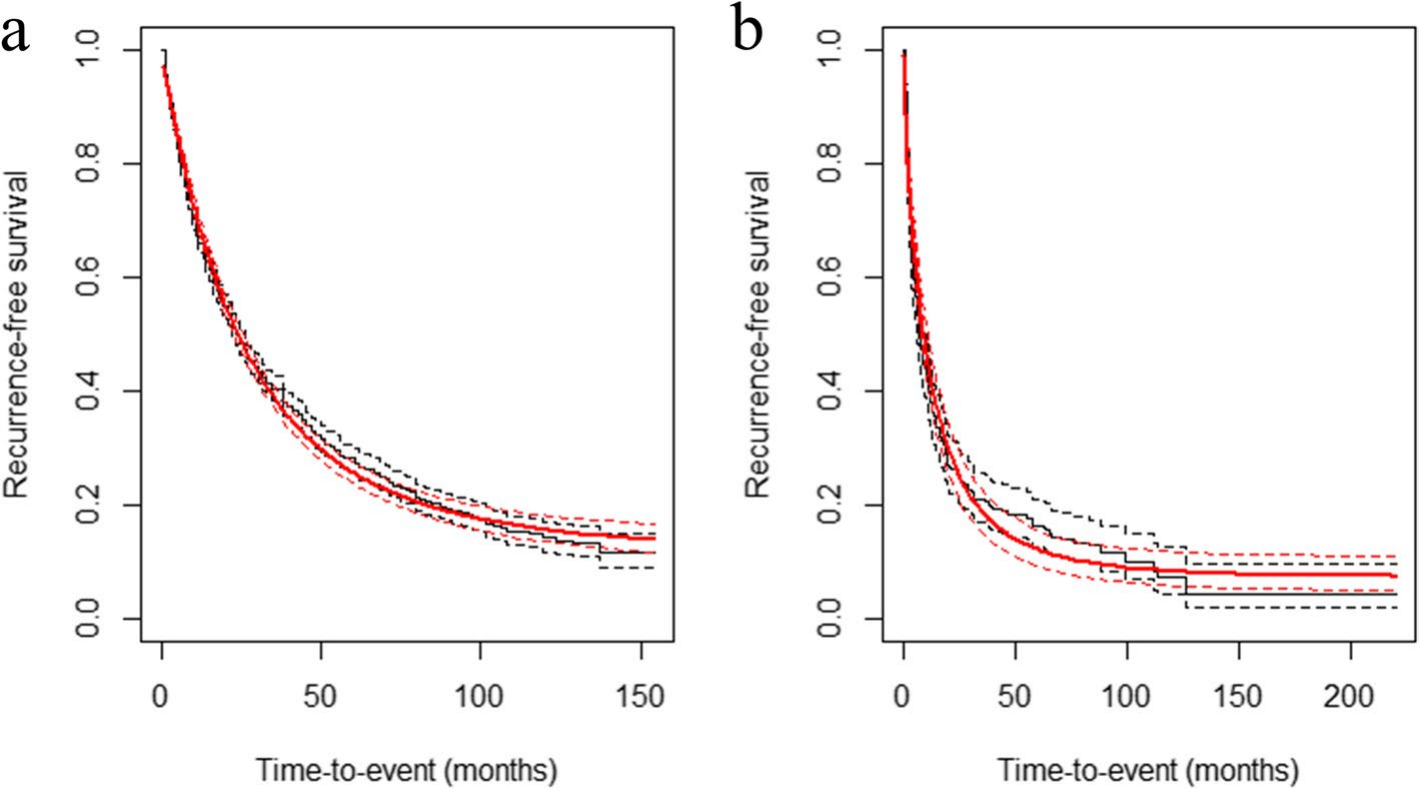
Predicted recurrence-free survival by cure model analysis in both the no-GVI group (**a**) and the mGVI group (**b**)

Pseudo-IPD of each study was reconstructed from the KM survival curves: 2,523 patients in the no-GVI group and 333 patients in the mGVI group [15, 24–26]. Analysis of this data using Kaplan–Meier methods demonstrated 1-, 3-, 5-, and 10-year survival rates of 66.1%, 40.3%, 28.1%, and 14.1% in the no-GVI group, respectively. The corresponding survival rates in the mGVI group were 39.2%, 20.8%, 16.3%, and 7.3%, respectively. The recurrence-free survival of patients in the mGVI group after SR was inferior to patients in the no-GVI group. (Fig. 2, *p*-value < 0.001). The probability of patients being cured by SR for HCC without GVI was 12.3% (95% CI, 9.8%–15.7%, Fig. 3a), and the time to statistical cure was 133.1 months (95% CI, 107.4–149.0). Among patients in the mGVI group who underwent SR, the probability of being cured was 7.3% (95% CI, 4.4%–11.2%, Fig. 3b), and the time to statistical cure was 113.8 months (95% CI, 90.5–124.6). From the non-mixture cure model results, a smaller proportion of patients were cured in the mGVI group than in the no-GVI group (HR, 1.81; 95% CI, 1.59 to 2.05; *P* < 0.001).

### Conditional survival probabilities analysis

The 5-year recurrence-free conditional survival (RFCS) probabilities and their changes in each additional year according to mGVI are outlined in Table 2. For patients in the no-GVI group and the mGVI group, the corresponding 5-year RFCS probabilities were 36.2% and 35.5% at the first year, and 50.2% and 44.8% at the fifth year, respectively. The increase in the 5-year RFCS probability was therefore greater in the no-GVI group (a 38.7% increase from 36.2% to 50.2%) than that of the mGVI group (a 26.2% increase from 35.5% to 44.8%). In particular, it should be noted that the 5-year RFCS probabilities of patients in the no-GVI group were steadily increasing. For patients in the mGVI group, an upward trend in the 5-year RFCS probabilities until the third year was observed, followed by a downward trend in the succeeding periods. One year after SR, the 5-year RFCS probability of patients with HCC presenting with mGVI was similar to that of those with HCC without GVI. The calculation of the standardized difference was also used to compare the 5-year RFCS probability differences observed at each year between groups. The value of the standardized difference (0.28 for 5-year recurrence-free survival rates) between two groups at the immediate postoperative period has been diminished. Over time, the value of the standardized differences in the 5-year RFCS probabilities observed between two groups were all less than 0.3. In other words, mGVI had an impact on the decreasing survival only for the first year.

**Table 2:** 5-year recurrence-free conditional survival probabilities in patients undergoing surgical resection for hepatocellular carcinoma presenting with minor gross vascular invasion or not

	Time elapsed since surgical resection (years)					
	0	1	2	3	4	5
Patients with HCC without GVI	28.1	36.2	40.7	44.9	48.6	50.2
Patients with HCC presenting with mGVI	16.3	35.5	49.6	55.8	54.9	44.8
Standarized difference	0.28	0.01	0.18	0.22	0.13	0.11

*HCC* Hepatocellular carcinoma, *mGVI* Minor gross vascular invasion

## Discussion

Although the standard practical guideline recommends systemic therapy for the treatment of HCC presenting with GVI, [7] the efficacy of SR when applied in clinical practice has been still investigated. Most of the clinical studies regarding this issue have been focused on the relative outcomes of SR for HCC with GVI compared with other treatment modalities, especially transarterial chemoembolization or sorafenib [13, 14]. These studies also showed that the comparative effectiveness of SR is more noticeable in treating HCC presenting with mGVI than that in HCC with major GVI. So, an analysis on the differences of the prognoses between patients with HCC presenting with mGVI and those with HCC without GVI could also contribute in the implementation of treatment policies. However, only a few studies have been conducted regarding this, especially about the difference of the long-term outcomes between these two groups. Therefore, the aim of this study is to compare the outcomes after SR for patients with HCC presenting with mGVI (mGVI group) and for patients with HCC without vascular involvement (no-GVI group), with regard to their long-term results. The cure model recently discussed and applied in various diseases for its capability to predict the probability of cure is of value; in our study, we used it to examine the cure fraction after SR among patients with HCC. The survival curve of patients with HCC after SR showed a plateau at the end. Thus, the cure model was used to analyze the data in both patient groups that showed cure fraction. We also compared the cure fraction in the mGVI group to those in the no**-**GVI group. The cure fraction of patients in the mGVI group after SR was also lower than that of patients in the no-GVI group (HR, 1.81; 95% CI, 1.59 to 2.05; *P* < 0.001). Nonetheless, the cure model analysis revealed that patients in the mGVI group had a meaningful cure fraction after SR (7.3% and 95% CI ranging from 4.4% to 11.2%). It means that a cure can be expected in around 7 percent of patients with HCC presenting with mGVI. To the best of our knowledge, no studies have directly compared the cure fraction of patients with HCC according to mGVI after SR.

In our study, using the CS probabilities, the clinical significance of mGVI and its changes over time in patients with HCC after SR was clearly shown. The values of mGVI estimated in the previous studies for the prediction of the survival rates were obtained at the initial presentation around the time of surgery [15, 24, 25]. Information about the changes in prognosis due to mGVI after a long-term survival period could not be provided. We could then empirically infer that cancer-related deaths would decrease over time even in patients with aggressive tumors, including mGVI, but evidence supporting this is lacking. This study is the first attempt to show the 5-year RFCS probability for patients with HCC presenting with mGVI who underwent SR, compared with that of those with HCC without GVI. We found that when conditioned in surviving one year after SR, the 5-year RFCS probability became nearly identical between the two groups. In other words, if patients with HCC presenting with mGVI undergoing SR survive more than 1 years, their prognosis would become the same as that of patients with HCC without GVI. Based on these results, we could reliantly deduce that the negative prognostic effect of mGVI would diminish after 1 year in the postoperative period.

It has been reported that GVI is one of significant prognostic factors for patients with HCC [27]. A recent study with a relatively large cohort sample analyzed the preoperative factors associated with a survival rate of more than 2 years after SR for HCC with GVI [28]. With the extent of GVI, an α-fetoprotein level over log 10 ug/L and clinically significant portal hypertension were found to be negative prognostic factors in this study. Another study indicated that preoperative thrombocytopenia, a symbol of portal hypertension, negatively impacted on postoperative recurrence of patients with HCC [29]. Furthermore, preoperative serum biomarkers such as α-fetoprotein and protein induced by vitamin K absence or antagonism factor II (PIVKA-II) have been already considered to be an independent prognostic factor for postoperative survival in patients with HCC [30]. When integrated into our findings, it is evident that SR can be selectively performed for patients with HCC presenting with mGVI in the absence of portal hypertension and the relatively low level of serum biomarkers. Additionally, sorafenib has been attention as an adjuvant treatment in patients undergoing SR for HCC [31]. It may be selectively applied to patients with HCC presenting with mGVI as an adjuvant therapy after SR.

CS probability analysis has been increasingly used to analyze the long-term effect of the tumor characteristics [32, 33]. For example, when comparing the efficacy of a certain treatment for patients in the advanced stage with that for patients in the early stage, patients in the advanced stage may have a higher risk during immediate treatment period but may have a much lower risk once they survive over a certain treatment period. The CS probability analysis could be particularly used to compare the difference of the immediate and late survival benefit after the treatment. This concept can be applied when determining the treatment for other critical diseases, such as other types of cancers, in patients with HCC presenting with mGVI. For example, reflecting on the results of this study, patients with HCC presenting with mGVI who survive without tumor recurrence more than one year after SR should be treated equally as those with HCC without GVI, because the life expectancy between the two groups is the same. These data may also help advise and inform patients about the necessary treatment options.

CS probability analysis can also be used in setting guidelines for the follow-up of patients with HCC presenting with mGVI undergoing SR. Unfortunately, the optimal postoperative follow-up strategy for advanced-stage HCC remains inconclusive. Current NCCN guidelines for HCC recommend every 3–6 months a follow-up on all patients in the first 2 years after SR, such as a subsequent biannual or annual follow-up [34]. No distinct approach to early and advanced stage patients is available at the moment. For instance, this interval may be too long for patients in the advanced stage, such as patients with HCC presenting with mGVI. However, assuming that the findings of this study are reliable, it doesn’t seem to be reasonable to make a difference of the follow-up interval according to vascular invasion status after the first postoperative year.

Our study has few limitations. First, it is based on a secondary analysis of the published primary data, which did not allow more detailed analyses. For example, the patients with HCC presenting with mGVI who underwent SR might have a better liver functional reserve than the average patients diagnosed with HCC. Other confounding variables, such as the level of tumor markers or the use of anti-viral agents, can affect the results. Unfortunately, a detailed description about them was not found. However, the presence and the extent of GVI are both independent predictors of the survival of patients with HCC in the primary studies. Second, in terms of the generalizability, we could only include three studies from Eastern countries, which do not represent the Western population. Third, this study is based on the collection of retrospective data. To verify the value of SR for HCC presenting with mGVI, compared with other therapeutic modalities such as sorafenib, prospective randomized trials are warranted.

## Conclusion

According to the results of our study, SR **is** able to cure HCC even in patients with HCC presenting with mGVI. Additionally, our findings showed that the 5-year RFCS probabilities after surviving the first postoperative year were essentially the same between patients with HCC presenting with mGVI and those with HCC without GVI. The findings of this study support the assertion that SR should be considered as a reliable therapeutic option for patients with HCC presenting with mGVI.

## Data Availability

Data sharing not applicable to this article as no datasets were generated or analysed during the current study.

## Abbreviations

CI: Confidence interval
CP: Child–Pugh
CS: Conditional survival
HCC: Hepatocellular carcinoma
HR: Hazard ratio
KM: Kaplan–Meier
mGVI: Minor gross vascular invasion
pseudo-IPD: Pseudo-individual patient data
RFCS: Recurrence-free conditional survival
SR: Surgical resection
GVI: Gross vascular invasion
